# Commencements

**Published:** 1893-07

**Authors:** 


### COMMENCEMENTS.

### Howard University—Dental Department.

The annual commencement of the Dental Department of the Howard University was held in connection with that of the Medical and Pharmaceutical departments at the Congregational Church, Washington, D. C., Wednesday evening, April 12, 1893.

The address to the graduates was delivered by Professor William H. Seaman, A.M., M.D.

The number of matriculates for the session was four.

The degree of D.D.S. was conferred by J. E. Rankin, D.D., LL D., President of the University, on L. N. Philipsen, of Louisiana.

### University of Pennsylvania—Department of Dentistry.

The fourteenth annual commencement of the Department of Dentistry of the University of Pennsylvania was held at the American Academy of Music, Philadelphia, Pennsylvania, on Wednesday, May 10, 1893.

The valedictory address was delivered by William F. Norris, M.D., professor of ophthalmology.

The number of matriculates for the session was one hundred and fifty-three.

The degree of D.D.S. was conferred on the following graduates by William Pepper, M.D., LL.D., Provost of the University.

| NAME | RESIDENCE |
|---|---|
| Walter B. Adams | Pennsylvania |
| Edward P. Betts | Pennsylvania |
| Frank J. Cahill | New Jersey |
| Elton C. Goodfellow | Pennsylvania |
| Antony Henneberg | Switzerland |
| Samuel Henneberg | Switzerland |
| Edward H. Hicks | Pennsylvania |
| Franklin M. Keffer | Pennsylvania |
| Charles F. Keim | Nebraska |
| Charles W. Neebe | South Africa |
| Guillermo Regardis | Venezuela |
| John R. Ricker | Texas |
| Jaky Rosen | Tennessee |
| William T. Sherman | Pennsylvania |
| Frank K. Stevens | Pennsylvania |
| A. Vergel de Dios | Philippine Islands |

June 16, 1892.

| NAME | RESIDENCE |
|---|---|
| Stephen H. Carey | Pennsylvania |
| Henry Elfers | Germany |
| Achille R. Nicodemi | Italy |
| M. N. Samaniego | Mexico |
| D. Everett Taylor | Connecticut |

Total, 21

### Columbian University—Dental Department.

The sixth annual commencement of the Dental Department of the Columbian University was held in connection with that of the Medical Department, at Albaugh's Opera House, Washington, D. C., on Thursday, May 4, 1893, at 2:30 P. M.

The address to the dental graduates was delivered by Professor Henry C. Thompson, D.D.S., and the valedictory by Edward G. Seibert, M.D.

The number of matriculates for the session was forty-four.

The degree of D.D.S. was conferred on the following graduates by J. C. Welling, LL.D., President of the University:

| NAME | RESIDENCE | NAME | RESIDENCE |
|---|---|---|---|
| Charles W. Appler | Maryland | Robert L. Nall | Kentucky |
| William N. Cogan | Dist. of Columbia | N. Willis Pomeroy | Dist. of Columbia |

Total, 4

### University of Buffalo—Dental Department.

The first annual commencement exercises of the Dental Department of the University of Buffalo, were held in connection with those of Medicine and Pharmacy, in Music Hall in the City of Buffalo, on the evening of May 2, 1893.

The number of matriculants for the session was forty-six.

The degree of D.D.S. was conferred on the following graduates, each having presented senior tickets from some reputable institution before joining the class:

| NAME | RESIDENCE | NAME | RESIDENCE |
|---|---|---|---|
| William Johnathan Crawford | Ohio | William Charles Smith | California |
| Edward Harry Lamport | New York | Daniel Hubbard Squire | New York |
| T. DeForest Phillips | New York | | |

Total, 5

### Indiana Dental College.

The fourteenth annual commencement of the Indiana Dental College was held on Thursday, March 30, 1893, at Indianapolis.

The number of matriculates for the session was sixty-six.

The degree of D.D.S. was conferred on the following persons:

| NAME | RESIDENCE | NAME | RESIDENCE |
|---|---|---|---|
| J. V. Voris | Brazil | C. D. Driscoll | Indiana |
| W. H. Upjohn | Indiana | | |

Total, 3

### University of Denver—Dental Department.

The fifth annual commencement exercises of the Dental Department of the University of Denver were held in connection with those of the Medical and Pharmaceutical Departments, on Wednesday evening, April 19, 1893.

The number of matriculates for the session was twelve.

Addresses were delivered by Rev. Dr. Duryea, Professors Henry Sewall, George J. Hartung, and James A. Sewall.

The degree of D.D.S. was conferred by Chancellor McDowell on Jesse Bert Macarey, of Colorado.

### Missouri Dental College.

The twenty-seventh annual commencement exercises of the Missouri Dental College, Dental Department of the Washington University, were held in connection with those of the Medical Department, at St. Louis, Missouri, on Tuesday, March 14, 1893.

The valedictory address was delivered by Dr. Frank R. Fry.

The degree of D.D.S. was conferred on the following graduates:

James Ambrose Hulen.

James Linton Jenkins.

William E. Weller.

Total, 3.

### Homœopathic Hospital College—Dental Department.

The second annual commencement of the Dental Department of the Homœopathic Hospital College, of Cleveland, Ohio, was held in connection with that of the other departments, on Wednesday, March 22, 1893.

The number of matriculates for the session was twelve.

The degree of D.D.S. was conferred on the following persons:

| NAME | RESIDENCE | NAME | RESIDENCE |
|---|---|---|---|
| John W. Wilson | Ohio | William M. Hutchison | Ohio |

Total 2

### University of Iowa—Dental Department.

The eleventh annual commencement of the Dental Department of the State University of Iowa was held at the Opera House, Iowa City, Iowa, on Thursday, March 16, 1893, at 9:30 A. M.

The number of matriculates for the session was one hundred and twenty.

The annual address was delivered by Professor Martin J. Wade, LL.B.

The degree of D.D.S. was conferred on the following persons by the President of the University, Charles A. Schaeffer, Ph.D.:

| NAME | RESIDENCE | NAME | RESIDENCE |
|---|---|---|---|
| Theodore Ashley | Minnesota | Joseph R. Kulp | Iowa |
| Benson W. Fordyce | Iowa | Henry S. Morgan | Iowa |
| Anna Hamilton Joy | Iowa | Samuel B. McNutt | Iowa |

Total, 6

### University of Minnesota—Dental Department.

The annual commencement excercises of the Dental department of the University of Minnesota were held, in connection with those of the other departments of the University, at Minneapolis, Minn., June, 1893.

The number of matriculates for the session was sixty-one.

The degree of D.M.D. (Doctor of Dental Medicine) was conferred on the following graduates:

| NAME: | RESIDENCE: | NAME: | RESIDENCE: |
|---|---|---|---|
| Caroline Augusta Edgar | Sauk Centre | George Silas Monson | St. Paul |
| Edward Haas, | St. Paul | Arthur Oscar Store | St. Paul |
| Mary Victorine Hartzell | Minneapolis | Henry Hurlburt Taylor | Minneapolis |
| Thos. Bradford Hartzell | Minneapolis | Oscar Albert Weiss | Hortonville, Wis. |
| Eugene Pollock Holmes | Faribault | Frank Noble Whittaker | Minneapolis |
| William Fred. Jewett | Minneapolis | George Wood Wood | Faribault |
| George Emery Means | Howard | | |

TOTAL 13

### Harvard University—Dental Department.

The annual commencement exercises of the Dental School of Harvard University were held in connection with those of the other departments of the University, in Sanders Hall, Cambridge, Mass, Wednesday, June 29, 1893, commencing at 10:30 A.M.

The degree of D.M.D. was conferred on fourteen graduates by Charles Wm. Eliot, LL.D., President of the University.

### College of Dental Surgery of the University of Michigan.

The eighteenth annual commencement of the Dental Department of the University of Michigan was held, in connection with the commencement exercises of the other departments, in University Hall, Ann Arbor, June 29th, 1893.

The number of matriculates for the session was one hundred and eighty-nine.

The annual commencement oration was delivered by Charles Dudley Warner, L.H.D.

The degree of D.D.S. was conferred on the following candidates by the President of the University, James B. Angell, LL.D.:

| NAME. | RESIDENCE. |
|---|---|
| Charles Wm. Adamson | Massachusetts |
| Alexander Robt. Allen | Michigan |
| Arthur William Ball | Michigan |
| Frank Irving Ball | Ohio |
| Frank Walter Boyer | Ohio |
| Herbert John Burke | Michigan |
| Charles Arthur Church | Michigan |
| William Jesse Clark | Oregon |
| William Arthur Conlan | Michigan |
| John Angell Cook | Michigan |
| Milton James Cook | Michigan |
| Harry Devillo Geiger | Michigan |
| Eugene Milton Graves | Oregon |
| James Grey | Michigan |
| Charles Augustus Hawley | Ohio |
| Marcellus Grant Hillman | Michigan |
| William Smith Hinckley | Michigan |
| Burdette C. Hinkley, D.D.S., Ohio College of Dental Surgery | Ohio |
| Elisha D. Hinkley, D.D.S., Ohio College of Dental Surgery | Ohio |
| Frank S. James | Michigan |
| Richard Davey Jones | Michigan |
| John William Kasbeer | Illinois |
| Herman Kreit | Michigan |
| Arthur Frederick Leuty | Michigan |
| George Blakesley Little | Iowa |
| Edward Ballard Lodge | Ohio |
| John Archibald McAllister | Utah |
| Robert Duncan McBride | Michigan |
| William McFarlane | Ontario, Canada |
| John M. McIlvain D.D.S., University of Maryland | Rhode Island |
| Jesse James McMullen | Iowa |
| Thomas Byron Mercer | Wisconsin |
| Walter Samuel Moore | Michigan |
| Mason Moyer | Indiana |
| Ethelwyn Phillips | England |
| Fred. M. Prettyman | Michigan |
| Weston A.V. Price | Ontario, Canada |
| Greenbury Albert Rawlings | N. Dakota |
| John George Schindler | Michigan |
| Frank Edward Seybold | Michigan |
| Louis N. Seymour, D.D.S., Philadelphia Dental College | New York |
| Edward G. Snodgrass, D.D.S., Ohio College of Dental Surgery | Ohio |
| John Francis Spring | Michigan |
| Milton Russell Stimson | Michigan |
| Burt Sidney Sutherland | Michigan |
| Sherman Hartwell Swift | Pennsylvania |
| Will Hamilton Van Deman | Ohio |
| John Hoffman Van den Berg | Michigan |
| Wm. Henry Van Iderstine | Michigan |
| Milton Tate Watson | Michigan |
| Will Lloyd Webster | Ohio |
| Henry Dudley Wilber | New York |
| Vernon Anderson Williams, D.D.S., Vanderbilt University | California |

Total, 53.

### The Louisville College of Dentistry.

The annual commencement exercises of the Louisville College of Dentistry (Dental Department of the Central University of Kentucky) took place on Tuesday evening, June 20th, at Masonic Temple, Louisville, Ky.

The valedictory oration was delivered by F. Clazie, of California. The degree of D.D.S. was conferred on the following graduates:

| Name: | Residence: | Name: | Residence: |
|---|---|---|---|
| F. Clazie | California | R. J. Hopkins | Kentucky |
| C. D. Coil | Texas | R. R. Keys | Indiana |
| M. J. Collins | California | C. L. Silvis | Illinois |

Total 6.

